# Molecular characterization of a naturally occurring intraspecific recombinant begomovirus with close relatives widespread in southern Arabia

**DOI:** 10.1186/1743-422X-11-103

**Published:** 2014-06-02

**Authors:** Mohammed A Al-Saleh, Ibrahim M Al-Shahwan, Judith K Brown, Ali M Idris

**Affiliations:** 1 Department of Plant Protection, King Saud University, Riyadh, Saudi Arabia; 2 School of Plant Sciences, The University of Arizona, Tucson, AZ, USA; 3 Center for Desert Agriculture, King Abdullah University of Science and Technology, Thuwal, Saudi Arabia

**Keywords:** Geminivirus, *Tomato leaf curl Sudan virus*, Virus variability, Whitefly vector

## Abstract

**Background:**

*Tomato leaf curl Sudan virus* (ToLCSDV) is a single-stranded DNA begomovirus of tomato that causes downward leaf curl, yellowing, and stunting. Leaf curl disease results in significant yield reduction in tomato crops in the Nile Basin. ToLCSDV symptoms resemble those caused by *Tomato yellow leaf curl virus*, a distinct and widespread begomovirus originating in the Middle East. In this study, tomato samples exhibiting leaf curl symptoms were collected from Gezira, Sudan. The associated viral genome was molecularly characterized, analyzed phylogenetically, and an infectious clone for one isolate was constructed.

**Findings:**

The complete genomes for five newly discovered variants of ToLCSDV, ranging in size from 2765 to 2767-bp, were cloned and sequenced, and subjected to pairwise and phylogenetic analyses. Pairwise analysis indicated that the five Gezira isolates shared 97-100% nucleotide identity with each other. Further, these variants of ToLCSDV shared their highest nucleotide identity at 96-98%, 91-95%, 91-92%, and 91-92% with the Shambat, Gezira, Oman and Yemen strains of ToLCSDV, respectively. Based on the high maximum nucleotide identities shared between these ToLCSDV variants from Gezira and other previously recognized members of this taxonomic group, they are considered isolates of the Shambat strain of ToLCSDV. Analysis of the complete genome sequence for these new variants revealed that they were naturally occurring recombinants between two previously reported strains of ToLCSDV. Finally, a dimeric clone constructed from one representative ToLCSV genome from Gezira was shown to be infectious following inoculation to tomato and *N. benthamiana* plants.

**Conclusion:**

Five new, naturally occurring recombinant begomovirus variants (>96% shared nt identity) were identified in tomato plants from Gezira in Sudan, and shown to be isolates of the Shambat strain of ToLCSDV. The cloned viral genome was infectious in *N. benthamiana* and tomato plants, and symptoms in tomato closely resembled those observed in field infected tomato plants, indicating the virus is the causal agent of the leaf curl disease. The symptoms that developed in tomato seedlings closely resembled those observed in field infected tomato plants, indicating that ToLCSDV is the causal agent of the leaf curl disease in Gezira.

## Background

Recent studies have shown that several tomato-infecting monopartite begomoviruses are prevalent in the Nile Basin, and in the southern region of the Arabian Peninsula, namely, Yemen and Oman [1]. Thus far, two species have been reported from the Nile Basin, *Tomato leaf curl Sudan virus* (ToLCSDV) and *Tomato yellow leaf curl virus* (TYLCV) [2,3], while three species have been recognized in the southern region of Arabia, *Tomato leaf curl Oman virus* (ToLCOMV), ToLCSDV and TYLCV [1,4]. This collective group of monopartite begomoviruses causes extensive damage to tomato (*Solanum lycopersicum* L.) crops in the arid and semi-arid southern part of the Arabian Peninsula and the Nile Basin [2,5].

Viruses in the genus, *Begomovirus* (family, *Geminiviridae*) are transmitted by the whitefly *Bemisia tabaci* (Genn.), a phloem-feeding aleyrodid in the Order: Hempitera. Begomoviruses are characterized by having a single-stranded circular DNA genome encapsidated in twinned icosahedral particles, and recently, have emerged to cause debilitating diseases to many species of dicotyledonous plants of agricultural importance [6]. They are widespread in uncultivated, endemic and naturalized plant species found in the tropics and subtropics worldwide [7,8]. The begomovirus genome is composed of either one (monopartite) or two components (bipartite), the latter being designated DNA-A and DNA-B components [9]. Monopartite and bipartite begomoviruses are present in the Eastern Hemisphere. In contrast, with the exception of the recently introduced *Tomato yellow leaf curl virus* (and its relatives) [10,11], and those associated with commercially traded ornamentals [8], and at times, sweet potato plants [12], only bipartite begomoviruses are found in the Western Hemisphere [6]. And, in a recent example reported from three locations in South America, the DNA-B component was shown to be dispensable for systemic infection [13,14], whereas, in another study involving infectious DNA-A component, no cognate DNA-B was detected [15].

The genome of monopartite begomoviruses contains certain genes that are functionally homologous to those encoded by the DNA-A component of bipartite genome type, encoding six overlapping open reading frames (ORF), two in the virion sense strand, and four in the complementary sense strand. Transcription of ORFs initiates from sequences in a non-coding region, referred to as the intergenic region (IR). This region also contains the begomoviral structurally conserved hairpin and origin of replication, and two or more virus replication associated protein (Rep)-binding motifs that are essential for viral genome replication [16]. Therefore, linkage of Rep and the IR would be expected during recombination, as well as preserved genome modularity, to enhance the prospects of producing fit and viable progeny [17].

In this study we report the molecular characterization of five new, naturally occurring, recombinant begomovirus variants arising from a putative intraspecific genomic exchange of sequences between two previously known strains of ToLCSDV, and demonstrate infectivity of a genomic clone for one of the variants.

## Methods

Leaf samples were collected from plants exhibiting yellow leaf curl and stunting symptoms growing in a commercial tomato field in Gezira, Sudan during the winter, 2011. Total DNA isolated from the symptomatic tomato plants was used as a template to amplify prospective begomoviral genomic components by rolling circle amplification technology (RCA) using the TempliPhi 100 Amplification Kit (GE Healthcare, Life Sciences, Piscataway, NJ, USA) as previously described [10,18]. The RCA product was digested to linearize the genome using *Eco*RI, cloned into pGEM7Zf + (Promega, Madison, WI) and completely sequenced. The DNA sequence was determined for each of five putative full-length begomoviral genomic clones, and analyzed using BLASTn algorithm to query the GenBank database (NCBI). The nucleotide (nt) sequence for the top 14 hits were included in pairwise comparison using the Species Demarcation Tool software (SDT v.1.0) [19] and phylogenetic analyses.

The aligned sequences were used to construct the phylogenetic trees using maximum Likelihood (ML) option available in PAUP* software [20], using the specific parameters, as described [4,21].

The sequence alignment used in the phylogenetic analyses was also used to identify potential recombinatorial fragments using the RDP2 software [22]. The default search parameters for recombination detection were employed using the highest acceptable probability (*P* value) of 0.01.

A recombinant plasmid (pGez3.1) cloned from the tomato sample Gezira 3, carrying a full-length begomoviral genome, was selected to construct a clone for infectivity using pGreen II [23]. The pGez3.1 clone was digested with *Eco*RI and *Bam*HI to release a 1191 bp fragment, and with *Eco*RI/*Cla*I to release a 2188 bp fragment. The fragments were ligated into pGreenII by inserting the *Cla*I-*Bam*HI fragments ultimately as a single insert, a process that was mediated by multi-fragment ligation [4] to obtain recombinant plasmid pG-Gez3. The pG-Gez3 was electroporated into *Agrobacterium tumefaciens* GV3101 and used to agro-infiltrate the leaves of *N. benthamiana*, cultivated tomato, *Solanum lycopersicon* cultivar M82, and wild tomato *S. cheesmaniae* accession LA0421, as previously described [4]. The infectivity tests were replicated three times. Viral DNA that accumulated in the subsequently developing (non-inoculated) leaves, following agroinoculation provided evidence of replication as well as local and long distance viral movement in the test plants. Total DNA was isolated from the newly expanded leaves of *N. benthamiana* and tomato that developed 7–12 days post-inoculation. Total DNA extracts were subjected to PCR analysis to ascertain begomoviral presence or absence, using ‘universal’ (core) coat protein primers [7]. The amplicons were cloned and sequenced.

## Results and discussion

**Figure 1:**
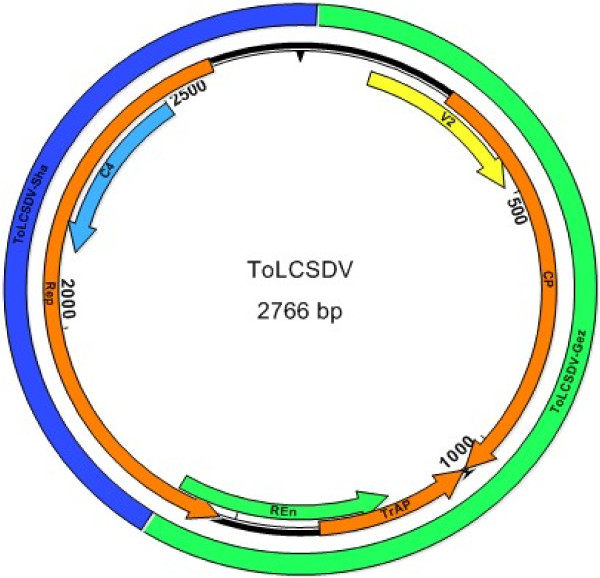
**Genomic map of ToLCSDV (inner circle) showing the six ORFs.** The origin of the recombinant fragments is indicated in the outer circle (from ToLCSDV-Sha[SD:Sha:96] in blue and from ToLCSDV-Gez[SD:Dez:96] in green). The recombinant fragments were identified using RDP, GENCONV and MaxChi.

The monopartite begomoviral genomes were cloned from the RCA-amplified products obtained using total DNA that was extracted from two different tomato plants (isolates Gez3 and Gez4) collected from Gezira. The plasmid vector contained ligated inserts (n = 5) that ranged in size from 2765-bp to 2767-bp. Sequence alignment followed by pairwise comparisons [19] revealed that the five clones shared 97-100% nt sequence identity. Inspection of the five genomic sequences revealed that they had features like other monopartite begomoviral genomes, based on the size and the characteristic organization of the six ORFs (V1, V2, C1, C2, C3 and C4), and the conserved IR [6,16] (Figure 1). The IRs contained one directly repeated sequence, or iteron, the TATA-box, and the stem-loop and nanonucleotide sequence, TAATATTAC, required for transcription and viral genome replication [24,25]. The sequences were used to search the NCBI GenBank database using BLASTn to identify the most closely related and several more distant taxa, which were selected and used as reference sequences (Additional file 1) for pairwise distance and phylogenetic analyses. The nt sequence comparisons revealed that the Gezira tomato isolates shared 88-96% nt identity with other ToLCSDV variants (Table 1), which until this report, were known to occur in the Nile Basin [2] and southern Arabia [1]. Further, the Gezira isolates shared 96-97% identity with the Shambat strain of ToLCSDV, also from the Nile Basin in Sudan.

**Table 1:** **Comparison of nucleotide sequence percentage identity for Gez3.1 of *Tomato leaf curl Sudan virus *** (ToLCSDV-Sha[SD:Gez3.1:11]) and other five recognized strains* of the ToLCSDV using Species Demarcation Tool (SDT v1.0)[19]

**Virus**	**Full-length**	**ORF**						**IR**	**Rep binding iteron**	**Iteron-related domain**
**[GenBank accession]**		**V1**	**V2**	**C2**	**C3**	**C1**	**C4**			
ToLCSDV	94	**99**	**99**	**98**	**98**	89	89	71	GGTGTAGTGGGGT	FKIN
[AY044139]										
ToLCSDV	**96**	97	93	95	94	**99**	**100**	**99**	GGTGTATCGGTAC	**FQIN**
[AY044137]										
ToLCSDV	92	97	93	87	88	89	87	86	TGTATATCGGTAC	FQIN
[JF919733]										
ToLCSDV	91	95	91	86	90	89	89	86	TGTATATCGGTAC	FQIN
[JF919731]										
ToLCSDV	92	97	93	86	87	90	87	81	GGTACATCGGTAC	FQIY
[JN591385]										
TYLCV	88	70	97	95	93	91	88	85	TGTATATCGGTAC	FQIN
[AY044138]										

*Different isolates of ToLCSDV from Gezira (AY044139), Shambat (AY044137), Yemen tomato (JF919731) and Yemen tobacco (JF919733) and *Tomato yellow leaf curl virus* from Gezira, Sudan (TYLCV). Comparisons are based on the complete genome sequence, and the nucleotide sequence of each individual open reading frame (ORF) (V1, V2, C1, C2, C3, C4) and the intergenic region (IR). The Rep-binding iteron and iteron-related domain for ToLCSDV-Sha[SD:Gez3.1:11] are identical to those for ToLCSDV-Sha[SD:Sha:96] strain (AY044137). Values in bold highlight the highest nucleotide sequence identities with ToLCSDV-Gez3.1 for each ORF or IR compared.

Based on the ICTV guidelines for species demarcation, at <91% nt identity [26], the Gezira tomato isolates are considered variants of ToLCSDV-Sha (Table 1). Even so, the Gezira isolates showed a range of divergence among the three main types of isolates that also group as the ToLCSDV species, including the Oman strain (ToLCSDV-Mir), at 91-92%, the Yemen strain (ToLCSDV-Ye), at 91-92%, and the ToLCSDV-Gez1 strain (also endemic to Sudan), at 91-95% nt identity.

Analysis of the ToLCSDV IR sequence revealed that the Rep-binding iterons, or the repeated ‘signature sequences’ associated with Rep-binding to initiate begomoviral replication, consisted of imperfect direct repeats (5’-GGTGTATCGGTAC-3’). These iterons were identical to those present in the sequence of the Shambat strain, as shown in Table 1. The iterons were located between the nt coordinates 2649 and 2661, or five nucleotides upstream of the TATA box of the *Rep* promoter. Comparative sequence analysis of the *Rep* gene of the ToLCSDV variants from Gezira, with previously recognized isolates and strains of this virus enabled visual detection of the conserved sequence, FQIN, and its predicted identification as the iteron-related domain (IRD), the predicted amino acid sequences that mediate iteron-Rep molecular interactions [16].

**Figure 2:**
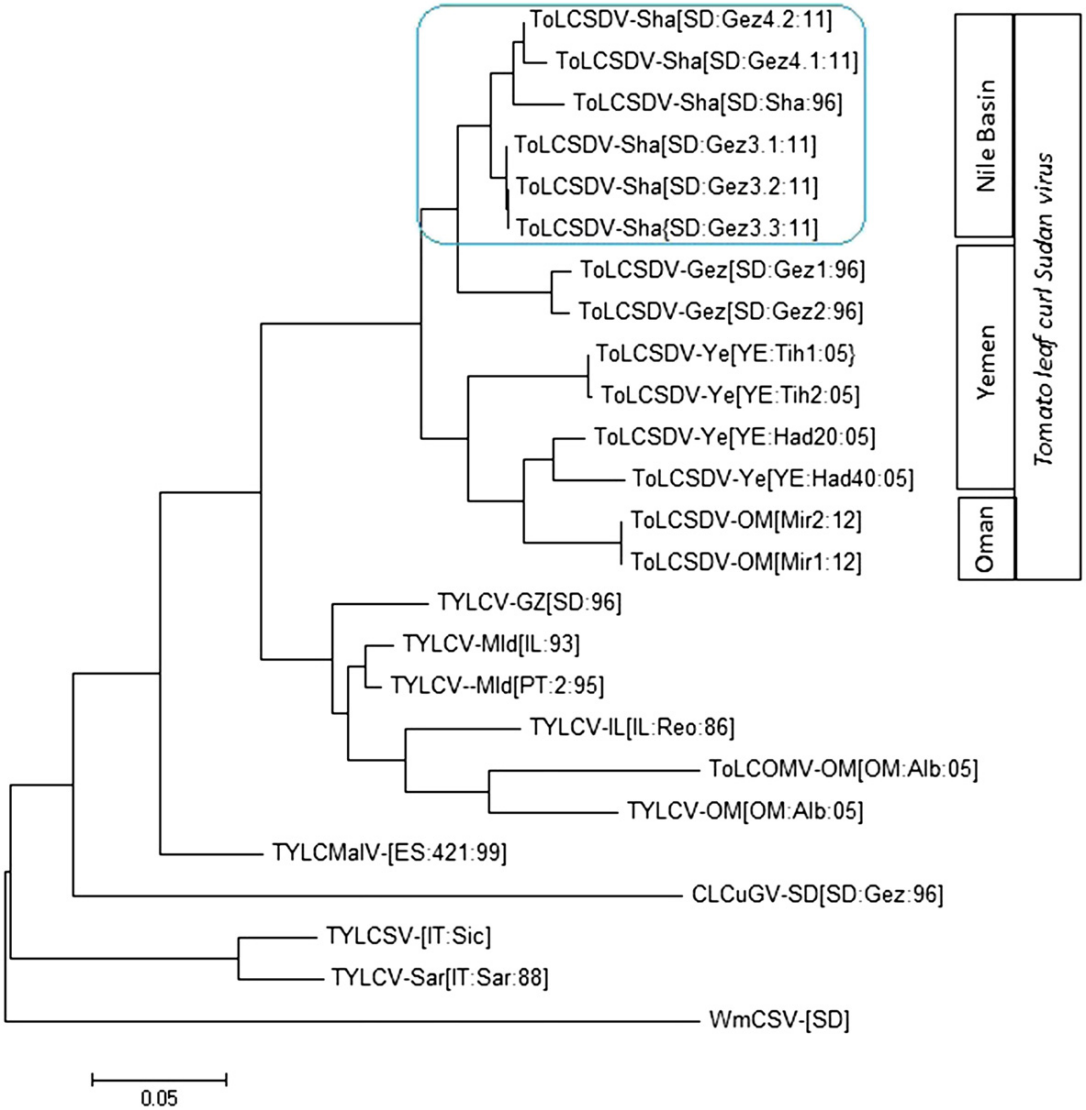
**Phylogenetic relationships for news variants of *Tomato leaf curl Sudan virus *and selected begomoviruses using the maximum likelihood algorithm available in Phylogenetic Analysis Using Parsimony, version 4.0.0b8 **[20]**.** The positions of the ToLCSDV isolates are shaded. Refer to additional table [see Additional file 1] for begomovirus acronyms and GenBank accession numbers and maximum parsimony phylogenetic tree [see Additional file 2] for bootstrap.

The results of the phylogenetic analysis of aligned begomoviral sequences indicated that all the isolates identified here, and previously recognized strains or variants of ToLCSDV, were members of a single clade (Figure 2; Additional file 2) also containing TYLCV and TYLCV-related begomoviruses extant in the Mediterranean region, North Africa, and the Arabian Peninsula. The five Gezira isolates (Gez3.1, Gez3.2 and Gez3.3 cloned from sample field Gez3 and Gez4.1 and Gez4.2 cloned from field sample Gez4) clustered with other members of the ToLCSDV-Sha strain clade. All of the ToLCSDV sequences grouped with a basis in geographical origin, e.g. either the Nile Basin or Asia, and the latter group was further divided by having an origin in Oman or Yemen. Previous reports have shown that geographical and/or other kinds of physical barriers can contribute importantly to the distribution of begomoviruses [6]. Generally, this group of viruses have been divided into two major groups extant in either the Eastern or Western Hemisphere, with the Eastern Hemisphere genomes (DNA-A or monopartite DNA component) being further separated into subclades, usually, also, exhibiting endemism either in the Far East, Southeast Asia, Africa, North Africa, and/or the Mediterranean region [2,3,21,27]. Based on these observations, the ToLCSDV species is likely endemic to the region extending from the Nile Basin to the southern region of the Arabian Peninsula [1].

Recombination analysis was carried out using the program suite available in RDP2 [14]. Three of these algorithms, RDP (*P* 3.772×10^−14^), GENCONV (*P* 3.604×10^−14^), and MaxChi (*P* 1.446×10^−10^), predicted that the genome of the herein five newly described ToLCSDV variants exhibit evidence of intraspecific recombination (Figure 1). The recombination event was confirmed by conducting an independent phylogenetic analysis of the recombinant fragments, which resulted in a shift in tree topology [see Additional file 3]. One predicted recombinant fragment (1199 nt) was located between the nt coordinates 1588–35, and based on nt sequence was most closely related to the Shambat strain, at 99% nt identity, but only 86% identical to the Gezira strain. The second predicated recombinant fragment (1566 nt in length) was located between the nt coordinates 36–1587, and its sequence was most closely related to the Gezira strain, at 99% nt identity, compared to its only 93% shared nt identity with the Shambat strain.

Seedlings of the cultivated tomato cv M18 and the wild tomato accession LA0421 [see Additional file 4], and of the model plant species, *N. benthamiana*, when agro-inoculated with the Shambat isolate of ToLCSDV (pG-Gez3), became systemically infected, based on characteristic viral symptom development. For *N. benthamiana,* the plants exhibited stunting, leaf curling, vein thickening, and stem deformation, which together resulted in a dwarfed and bushy appearance [see Additional file 5] 7–8 day post-inoculation (DPI). The ToLCSDV-infected ‘wild’ tomato accession LA0421, exhibited upward curling of the old leaflets, reduced size of new leaflets, and mild vein yellowing, 10–11 DPI. In contrast, the cultivated tomato developed severe curling of new leaflets 11–12 DPI, the symptom phenotype most commonly observed in field infected tomato plants. ToLCSDV presence or absence was confirmed by PCR amplification, and cloning and DNA sequencing of the core region of the viral coat protein (data not show) from total plant DNA extracted from newly developing leaves 12 DPI, thereby fulfilling Koch’ postulates for this isolate. In comparison, virus-like symptoms were not observed in the mock-inoculated seedlings of *N. benthamiana*, LA0421, or cv M18.

The leaf curl disease of tomato in Gezira, Sudan (the Nile Basin) has long been associated with begomoviral presence, and isolates of ToLCSDV have been cloned and sequenced from tomato plants collected there as early as 1996 [2]. However, this is the first report of the cloning and sequencing of multiple begomoviral variants from tomato plants exhibiting leaf curl symptoms, and of the demonstrated infectivity of the cloned genome of a representative isolate of ToLCSDV in Gezira, Sudan. Thus, at the time of sample collection, ToLCSDV can be considered the causal agent of the leaf curl disease occurring in tomato crops in Gezira, Sudan. The infectious clone required no associated satellite-like molecule for the development of wild type symptoms in tomato. The development of this infectious ToLCSDV clone provides a whitefly-free approach for rapid resistance screening of tomato germplasm and other solanaceous hosts of the virus. It also provides information inherent in the viral genome sequence that can be used to devise virus- or sequence-specific targets for RNAi-mediated resistance.

## Competing interests

The authors declare that they have no competing interests.

## Authors’ contributions

AMI collected the samples, cloned and sequenced the new virus variant. All of the authors were awarded funding for this research project, participated in aspects of the experimental design and manuscript preparation including discussion and editing. All of the authors have read and approved the final version of the manuscript.

## Supplementary Material

Additional file 1 **Begomovirus name and acronym **[16]**, and corresponding GenBank accession number for each.** Click here for file

Additional file 2 **Phylogenetic relationships for news variants of *Tomato leaf curl Sudan virus *** **and selected begomoviruses using the maximum parsimony algorithm available in Phylogenetic Analysis Using Parsimony, version 4.0.0b8 **[20]**.** The positions of the ToLCSDV isolates are shaded. Refer to additional table [see Additional file 1] for begomovirus acronyms and GenBank accession numbers. Click here for file

Additional file 3 **Phylogenetic relationships for ToLCSDV-Sha[SD:Gez3.1:11] and other ToLCSDV strains using maximum parsimony (MP) analysis to corroborate the RDP-predicted sites of recombination.** Trees show MP results for the **(A)** fragment (nt 1588–35), and **(B)** fragment (nt 36–1587). The bootstrap values are shown in major nodes. Arrows indicate shift of predictive parent positions. Refer to additional table [Additional file 1] for begomovirus acronyms and GenBank accession numbers. Click here for file

Additional file 4 **Symptoms of *Tomato leaf curl Sudan virus *** **(ToLCSDV-Sha[SD:Gez3.1:11]) in virus-infected tomato plants.** The tomato cultivar, M18 (Ali Mahjoub, KAUST, Thuwal, Saudi Arabia), was used for inoculation experiments. Treatments were: **(A)** ToLCSDV-inoculated, **(B)** mock-inoculated plant. Wild type accession LA0421 (Ali Mahjoub, KAUST, Thuwal, Saudi Arabia), **(C)** ToLCSDV-inoculated plant, and **(D)** mock-inoculated plant. Click here for file

Additional file 5 **Symptoms of *Tomato leaf curl Sudan virus* (ToLCSDV) in *Nicotiana benthamiana* plants. (A)** ToLCSDV-inoculated plant, **(B)** close-up of a leaf lower surface of ToLCSDV-inoculated plant, **(C)** mock-inoculated plant, and **(D)** close-up of a leaf lower surface of mock-inoculated plant. Click here for file
