# Supplementary material for: Molecular characterization of a naturally occurring intraspecific recombinant begomovirus with close relatives widespread in southern Arabia
Source: Virol J. 2014 Jun 2;11:103. doi: 10.1186/1743-422X-11-103 (PMC4071017; doi:10.1186/1743-422X-11-103)
Supplement: Additional file 1 — Begomovirus name and acronym [16], and corresponding GenBank accession number for each. [file 1743-422X-11-103-S1.docx]

Begomovirus name and acronym [11], and corresponding GenBank accession number for each.

| **Begomovirus** | **Acronym** | **GenBank accession number** |
| --- | --- | --- |
| Cotton leaf curl Gezira virus-Sudan [Sudan:Gezira] | CLCuGeV-SD[SD:Gez:96] | AF260241 |
| Tomato leaf curl Oman virus-Oman[Oman:Al-Batinah:2005] | ToLCOMV-OM[OM:Alb:05] | FJ956700 |
| Tomato leaf curl Sudan virus- Oman[Oman:Mirbat 2:2012] | ToLCSDV-OM[OM:Mir2:12] | JN591386 |
| Tomato leaf curl Sudan virus- Yemen[Yemen:Hadramut20:2005] | ToLCSDV-Ye[YE:Had20:05] | JF919733 |
| Tomato leaf curl Sudan virus- Yemen[Yemen:Hadramut40:2005] | ToLCSDV-Ye[YE:Had40:05] | JF919734 |
| Tomato leaf curl Sudan virus- Yemen[Yemen:Tihama5:2005] | ToLCSDV-Ye[YE:Tih5:05] | JF919732 |
| Tomato leaf curl Sudan virus-Gezira [Sudan:Gezira1:1996] | ToLCSDV-Gez[SD:Gez1:96] | AY044137 |
| Tomato leaf curl Sudan virus-Gezira [Sudan:Gezira2:1996] | ToLCSDV-Gez[SD:Gez2:96] | GU180085 |
| Tomato leaf curl Sudan virus-Oman[Oman:Mirbat 1:2012] | ToLCSDV-OM[OM:Mir1:12] | JN591385 |
| Tomato leaf curl Sudan virus-Shambat[Sudan:Shambat:1996] | ToLCSDV-Sha[SD:Sha:96] | AY044139 |
| Tomato leaf curl Sudan virus-Yemen[Yemen:Tihama1:2005] | ToLCSDV-Ye[YE:Tih1:05] | JF919731 |
| Tomato yellow leaf curl Malaga virus-[Spain:421:1999] | TYLCMalV-[ES:421:99] | AF271234 |
| Tomato yellow leaf curl Sardinia virus-Sardinia[Italy:Sardinia:1988] | TYLCSV-Sar[IT:Sar:88] | X61153 |
| Tomato yellow leaf curl Sardinia Virus-Sicily[Italy:Sicily] | TYLCV-Sic[IT-Sic] | Z28390 |
| Tomato yellow leaf curl virus-Gezira [Sudan:1996] | TYLCV-Gez[SD:96] | AY044138 |
| Tomato yellow leaf curl virus-Israel [Israel:Rehovot:1986] | TYLCV-IL[IL:Reo:86] | X15656 |
| Tomato yellow leaf curl virus-Mild [Israel:1993] | TYLCV-Mld[IL:93] | X76319 |
| Tomato yellow leaf curl virus-Mild [Portugal:1995] | TYLCV-Mld[PT:95] | AF105975 |
| Tomato yellow leaf curl virus-Oman [Oman:Al-Batinah:2005] | TYLCV-OM[OM:Alb:05] | DQ644565 |
| Watermelon chlorotic stunt virus-[Sudan] | WmCSV-[SD] | AJ245650 |
