# Supplementary material for: Molecular characterization of a naturally occurring intraspecific recombinant begomovirus with close relatives widespread in southern Arabia
Source: Virol J. 2014 Jun 2;11:103. doi: 10.1186/1743-422X-11-103 (PMC4071017; doi:10.1186/1743-422X-11-103)
Supplement: Additional file 2 — Phylogenetic relationships for news variants of Tomato leaf curl Sudan virus and selected begomoviruses using the maximum parsimony algorithm available in Phylogenetic Analysis Using Parsimony, version 4.0.0b8 [20]. The positions of the ToLCSDV isolates are shaded. Refer to additional table [see Additional file 1] for begomovirus acronyms and GenBank accession numbers. [file 1743-422X-11-103-S2.pptx]

## Slide 1
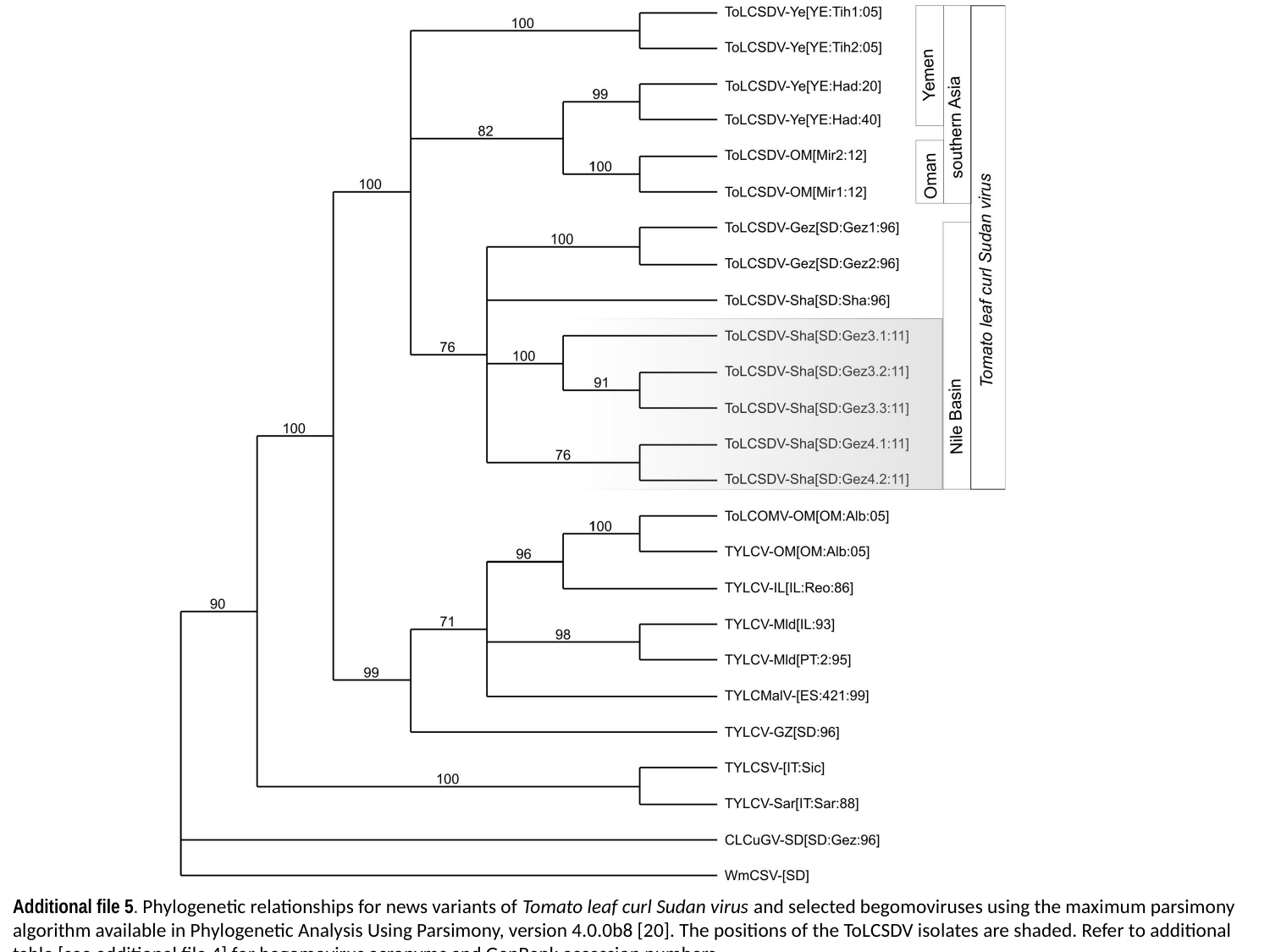

# Figure 2.
Additional file 5. Phylogenetic relationships for news variants of Tomato leaf curl Sudan virus and selected begomoviruses using the maximum parsimony algorithm available in Phylogenetic Analysis Using Parsimony, version 4.0.0b8 [20]. The positions of the ToLCSDV isolates are shaded. Refer to additional table [see additional file 4] for begomovirus acronyms and GenBank accession numbers.
