# Supplementary material for: Molecular characterization of a naturally occurring intraspecific recombinant begomovirus with close relatives widespread in southern Arabia
Source: Virol J. 2014 Jun 2;11:103. doi: 10.1186/1743-422X-11-103 (PMC4071017; doi:10.1186/1743-422X-11-103)
Supplement: Additional file 3 — Phylogenetic relationships for ToLCSDV-Sha[SD:Gez3.1:11] and other ToLCSDV strains using maximum parsimony (MP) analysis to corroborate the RDP-predicted sites of recombination. Trees show MP results for the (A) fragment (nt 1588–35), and (B) fragment (nt 36–1587). The bootstrap values are shown in major nodes. Arrows indicate shift of predictive parent positions. Refer to additional table [Additional file 1] for begomovirus acronyms and GenBank accession numbers. [file 1743-422X-11-103-S3.pptx]

## Slide 1
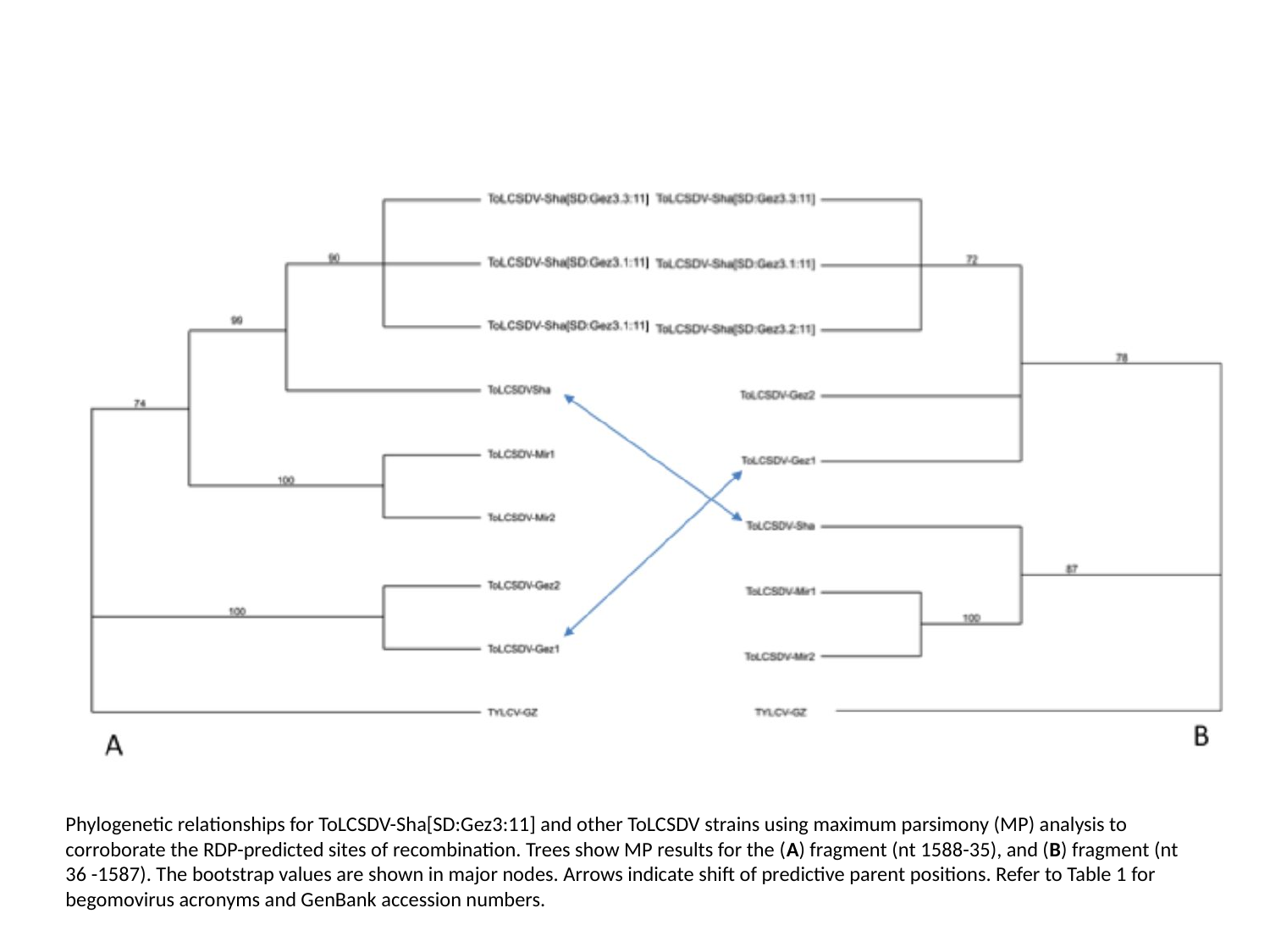

# Phylogenetic relationships for ToLCSDV-Sha[SD:Gez3:11] and other ToLCSDV strains using maximum parsimony (MP) analysis to corroborate the RDP-predicted sites of recombination. Trees show MP results for the (A) fragment (nt 1588-35), and (B) fragment (nt 36 -1587). The bootstrap values are shown in major nodes. Arrows indicate shift of predictive parent positions. Refer to Table 1 for begomovirus acronyms and GenBank accession numbers.
