# Supplementary material for: Molecular characterization of a naturally occurring intraspecific recombinant begomovirus with close relatives widespread in southern Arabia
Source: Virol J. 2014 Jun 2;11:103. doi: 10.1186/1743-422X-11-103 (PMC4071017; doi:10.1186/1743-422X-11-103)
Supplement: Additional file 4 — Symptoms of Tomato leaf curl Sudan virus (ToLCSDV-Sha[SD:Gez3.1:11]) in virus-infected tomato plants. The tomato cultivar, M18 (Ali Mahjoub, KAUST, Thuwal, Saudi Arabia), was used for inoculation experiments. Treatments were: (A) ToLCSDV-inoculated, (B) mock-inoculated plant. Wild type accession LA0421 (Ali Mahjoub, KAUST, Thuwal, Saudi Arabia), (C) ToLCSDV-inoculated plant, and (D) mock-inoculated plant. [file 1743-422X-11-103-S4.pptx]

## Slide 1
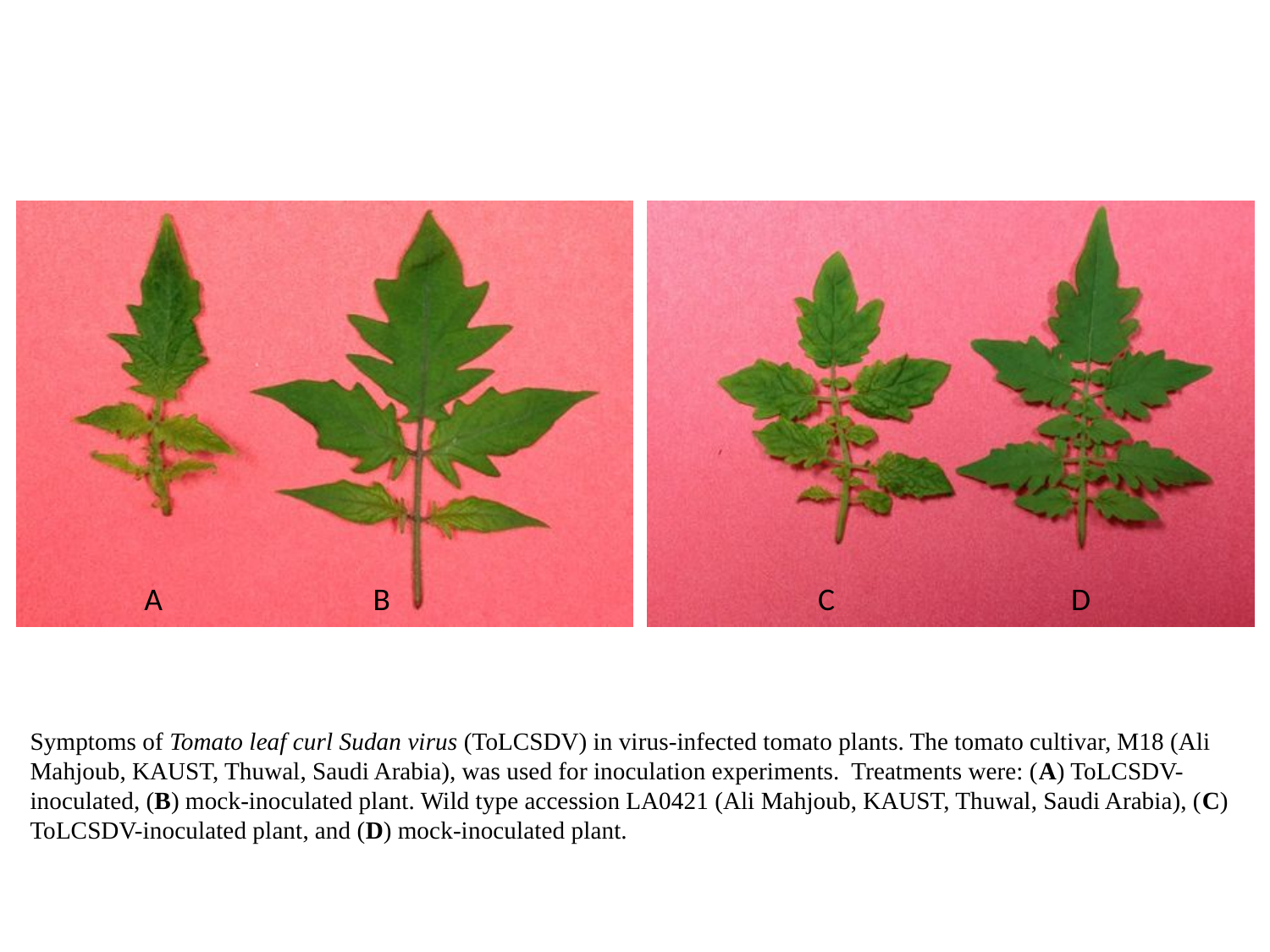

A
B
C
D
Symptoms of Tomato leaf curl Sudan virus (ToLCSDV) in virus-infected tomato plants. The tomato cultivar, M18 (Ali Mahjoub, KAUST, Thuwal, Saudi Arabia), was used for inoculation experiments. Treatments were: (A) ToLCSDV-inoculated, (B) mock-inoculated plant. Wild type accession LA0421 (Ali Mahjoub, KAUST, Thuwal, Saudi Arabia), (C) ToLCSDV-inoculated plant, and (D) mock-inoculated plant.
