# Supplementary material for: Molecular characterization of a naturally occurring intraspecific recombinant begomovirus with close relatives widespread in southern Arabia
Source: Virol J. 2014 Jun 2;11:103. doi: 10.1186/1743-422X-11-103 (PMC4071017; doi:10.1186/1743-422X-11-103)
Supplement: Additional file 5 — Symptoms of Tomato leaf curl Sudan virus (ToLCSDV) in Nicotiana benthamiana plants. (A) ToLCSDV-inoculated plant, (B) close-up of a leaf lower surface of ToLCSDV-inoculated plant, (C) mock-inoculated plant, and (D) close-up of a leaf lower surface of mock-inoculated plant. [file 1743-422X-11-103-S5.pptx]

## Slide 1
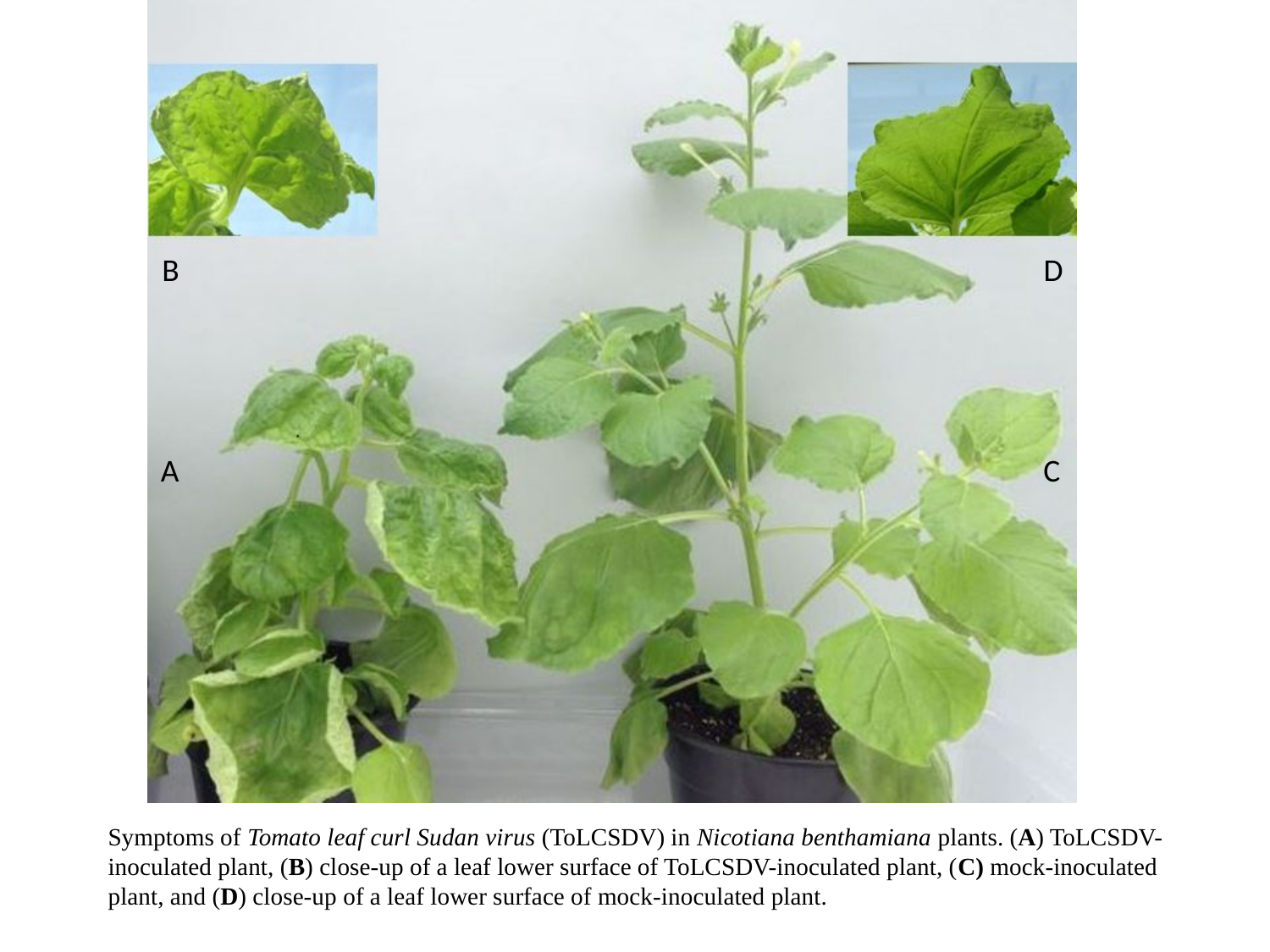

B
D
A
C
#
Symptoms of Tomato leaf curl Sudan virus (ToLCSDV) in Nicotiana benthamiana plants. (A) ToLCSDV-inoculated plant, (B) close-up of a leaf lower surface of ToLCSDV-inoculated plant, (C) mock-inoculated plant, and (D) close-up of a leaf lower surface of mock-inoculated plant.
